# Inter-molecular epitope spreading does not lead to extension of autoimmunity beyond target tissue in autoimmune glomerulonephritis

**DOI:** 10.1371/journal.pone.0202988

**Published:** 2018-08-28

**Authors:** April Ross, Jean Wu, Colin Carlock, William Glass, Ya-Huan Lou

**Affiliations:** 1 Department of Diagnostic Sciences, School of Dentistry, University of Texas Health Science Center at Houston, Houston, Texas, United States of America; 2 Department of Pathology, McGoven Medical School, University of Texas Health Science Center at Houston, Houston, Texas, United States of America; Duke University School of Medicine, UNITED STATES

## Abstract

Inter-molecular epitope spreading during autoimmune pathogenesis leads to generation of new pathogenic epitopes on other autoantigens beyond the original one. It raises an important question as whether autoimmunity extends beyond the target tissues if new epitopes are on the molecules shared with other tissues. This study is aimed addressing this question in a rat anti-glomerular basement membrane (GBM) glomerulonephritis model induced by a T cell epitope of glomerulus-specific collagen4α3. We have demonstrated inter-molecular B cell epitope spreading. Four novel epitopes were first identified by screening a phage display random peptide library against autoantibodies isolated from the GBM of immunized rats. All four epitopes were derived from GBM proteins with three from laminins and one from collagen4α4. Three out of four synthetic peptides were nephritogenic. Importantly, two peptides from lamininα1 and lamininβ1, respectively, induced severe inflammation in glomeruli but not in the interstitial tissues, despite the presence of more abundant laminins in the tubular basement membranes. Our study suggests that surrounding tissues may display a lower or altered susceptibility to autoimmune inflammation. Thus, preventing extension of autoimmune inflammation beyond the original target tissue.

## Introduction

Autoreactive T cells and B cells may be activated through internal auto-immunization in individuals with deficiencies in self-tolerance mechanisms, or through infections [1, 2]. Tissue damage caused by the original inflammation in the target tissues, in turn, leads to the release of large quantities of diverse autoantigens. *Do released autoantigens induce more autoimmunization due to defective host self-tolerance mechanisms*? The role of “leaking” autoantigens in causing autoimmunization was demonstrated over the past two decades. Such autoimmunization leads to the activation of additional autoreactive T and B cells. This phenomenon, also termed “epitope spreading”, was first described in the experimental autoimmune encephalomyelitis (EAE) mouse model, in which autoimmunity spreads to a cryptic T cell epitope on the same autoantigen [3]. Shortly thereafter, spreading of T cell-autoimmunity to autoreactive B cell epitopes was discovered [4, 5]. Since then, epitope spreading has been demonstrated in many animal models [6–13]. Anti-glomerular basement membrane (GBM) glomerulonephritis (GN) and lupus GN are of an autoimmune nature [14, 15]. Generation of new autoantibodies to diverse autoantigens beyond the original one has been reported in GNs [8, 9, 12, 13]. Thus, epitope spreading can occur inter-molecularly. It raises several important questions. First, whether new epitopes on the other autoantigens are pathogenic? If so, does inter-molecular epitope spreading causeextension of autoimmunity beyond the original target organ, since the new pathogenic epitopes may be on the autoantigens shared with surrounding tissues? However, in the majority of human autoimmune diseases, as well as in animal models, autoimmune damage is usually restricted to certain tissue(s) despite inter-molecular epitope spreading. Therefore, an unknown mechanism must exist to limit extension of pathogenic autoimmunity to other tissues due to epitope spreading.

We have developed a rat anti-GBM GN model, in which, severe GN is induced in Wistar Kyoto (WKY) rats by immunization with a single T cell epitope pCol4α3(28–40) of glomeruli-specific autoantigen collagen 4α3 [16, 17]. Immunization with this T cell epitope also results in production of diverse anti-GBM autoantibodies to autoantigens beyond collagen4α3 [8, 16]. We further demonstrated that activation of autoreactive B cells is initiated in renal draining lymph nodes [18]. This study was designed to test whether newly generated B cell epitopes were able to cause autoimmunity outside of the target tissue glomeruli. We first identified four new B cell epitopes in GBM proteins other than collagen 4α3 using isolated anti-GBM autoantibodies. Three of them were highly nephritogenic. Interestingly, two pathogenic peptides, which were derived from common basement membrane proteins laminins, caused significant inflammation in glomeruli, but not in tubules or the interstitium. Thus, each tissue probably has a special susceptibility to autoimmunity, which may prevent spread of autoimmunity beyond the target tissues.

## Materials and methods

### Induction and evaluation of anti-GBM glomerulonephritis in rats

All procedures involving animals in this study were approved by The Animal Welfare Committee, which is the Institutional Animal Care and Use Committee (IACUC) for the University of Texas Health Science Center at Houston (UTHealth). Female WKY rats (4–6 weeks of age) were purchased from Harlan Laboratories (Indianapolis, IN), and allowed to acclimate for three days in the animal facility at the University of Texas Health Science Center at Houston. For induction of anti-GBM GN, nephritogenic peptide pCol(28–40), or other identified epitopes (0.15 μmol) were first emulsified in CFA for immunization [16]. Rats were immunized by injection in one hind footpad and in the base of the tail with mixture of CFA and peptide, or CFA (as a negative control). Immunized rats were monitored daily for infection or inflammation at the injection sites. Rats would have been euthanized had open-skin ulcers occurred at injection sites, or had rats failed to reach foods or water, but neither problems occurred in this study. Each rat was monitored daily for GN progression by albuminuria. Two μls of randomly sampled urine was loaded onto 12.5% SDS-PAGE, and albumin concentration was determined by comparison to a bovine serum albumin standard [17]. Immunized rats were euthanized as planned at day 41 for isolation of autoantibody, or 45 for all other purposes. Renal tissues were fixed in Bouin’s solution, or snap-frozen in liquid nitrogen. GN was evaluated by H&E and PAS staining of fixed renal tissues. Severity of GN for each was expressed as the percentage of glomeruli with hypercellularity and necrosis. Snap-frozen tissues were used for immunofluorescence quantitation of macrophage subsets, and for detection and isolation of GBM-bound IgG autoantibodies. In total, 85 WKY rats were used for this study. Among them, 30 rats were used for isolation of anti-GBM autoantibodies, and 55 for induction of anti-GBM GN with different peptides.

### Isolation of glomeruli and elution of anti-GBM autoantibodies

Kidneys from rats immunized with pCol4α3(28–40) were first observed for presence of IgG type anti-GBM autoantibody binding by direct immunofluorescence. Usually, 10 pairs of kidneys were selected for one Ab elution experiment. Glomeruli were first isolated from those kidneys showing intense binding of IgG anti-GBM autoantibody following an established method [8]. Briefly, kidneys were placed in DMEM. The cortex was minced, and ground through a 75μm steel mesh. The material that passed through the mesh contained the glomeruli; the crude glomeruli was collected and filtered again through a 40μm filter to exclude other tissue debris. After repeated centrifugations and gravity settlement, purified glomeruli were further washed 8 times with cold PBS to eliminate contaminant IgG. Resultant glomeruli were briefly washed by citric acid/sodium citrate buffer (0.1M, pH 3.2), and incubated with 1ml of the same buffer for 10 minutes to elute anti-GBM autoantibody. Supernatant, which contained eluted autoantibody, was collected and immediately neutralized with 1M NaOH to pH 8.0. This elution process was repeated three times to ensure maximal yield of anti-GBM autoantibody. Pooled eluate was dialyzed against ammonium bicarbonate buffer at 4°C for overnight, and lyophilized. A small portion of eluate was pre-removed after neutralization and used for measuring IgG and anti-GBM autoantibody activity. The lyophilized eluate powder was re-suspended with cold PBS and retested for Ab activities, and aliquots were kept at -80°C. Total three isolations were performed and thirty immunized WKY rats were used.

### Epitope mapping with a phage display random peptide library

To identify epitopes recognized by the eluted anti-GBM autoantibody, a combinatorial library peptide (12-mers) phage display kit was utilized (Ph.D.™ 12mer Phage Display Library, New England Biolabs, MA) following manufacturer’s protocol for solution phase panning with affinity bead capture. Briefly, 45μg eluted anti-GBM autoantibody was pre-incubated with 50μl Dynabeads® Protein G (Novex, Life Technologies, Carlsbad, CA) for complete bead saturation. The Ab-saturated beads were then incubated with 2x10^11^ pfu of the M13 phage library with a library complexity of 2x10^9^. The bound phages were collected by magnetic field and eluted by incubation for 10 minutes with 1ml of glycine elution buffer (0.2M Glycine-HCl, pH2.2, 1mg/ml BSA). The eluate was neutralized immediately with 150 μl of 1M Tris-HCl, pH 9.1. An aliquot of eluate was used for titering phage on LB/IPTG/XGAL plates. A 1:100 dilution of the remaining eluate was amplified overnight by inoculation of LB+Tet with ER2738 *E*. *coli* at 37°C with shaking, followed by centrifugation for 10 minutes at 12,000 *g* at 4°C. A 1/6 volume of 20% PEG/2.5M NaCl added to the supernatant, which contained the phages, and then allowed to settle overnight at 4°C for precipitation of phages. Precipitated phages were collected by centrifugation at 12,000 *g* at 4°C for 15 minutes, and re-suspended in 1 ml TBS. An additional round of phage precipitation was performed and allowed to incubate on ice for 1 hour. After 2 brief centrifugations, the pellet was re-suspended with a final volume of 200 μl of TBS, which was then used for second round panning by the same batch of anti-GBM autoantibody. After three rounds of panning, randomly selected phage plaques were amplified for Western blot against the same anti-GBM autoantibody or for isolation of DNA for sequencing the insert in gene *pIII*. A phage clone that had a positive reactivity to the Ab was further tested against normal rat IgG by Western blots. Only those, which were positive to anti-GBM autoantibody but negative to rat IgG, were sequenced for their insert DNA (SeqWright, Houston, TX) using the 96 *g*III sequencing primer (5’- ^HO^CCC TCA TAG TTA GCG TAA CG-3’).

### Immunofluorescence

Direct immunofluorescence was used to detect and titter IgG type GBM-bound autoantibodies. Briefly, kidneys of immunized rats were snap-frozen and frozen sections were cut. Serially diluted FITC-anti-rat IgG Ab was incubated with the sections. Indirect immunofluorescence was performed to titter eluted anti-GBM autoantibodies. The eluted Abs were serially diluted and incubated with normal WKY rat or human kidney sections, followed by incubation with a FITC-anti-rat IgG Ab. De-identified human kidney tissues were obtained from BioBank UT-Health without any identifiable information. Frozen kidneys from immunized rats were used for detecting subsets of macrophages with combination of Abs to RT1B and ED1 (CD68), or for detecting fibrotic tissue with anti-collagen1α1 Ab following established methods [19].

## Results

### Purification of anti-GBM autoantibodies from pCol4α3(28–40)-immunized WKY rats

**Fig 1:**
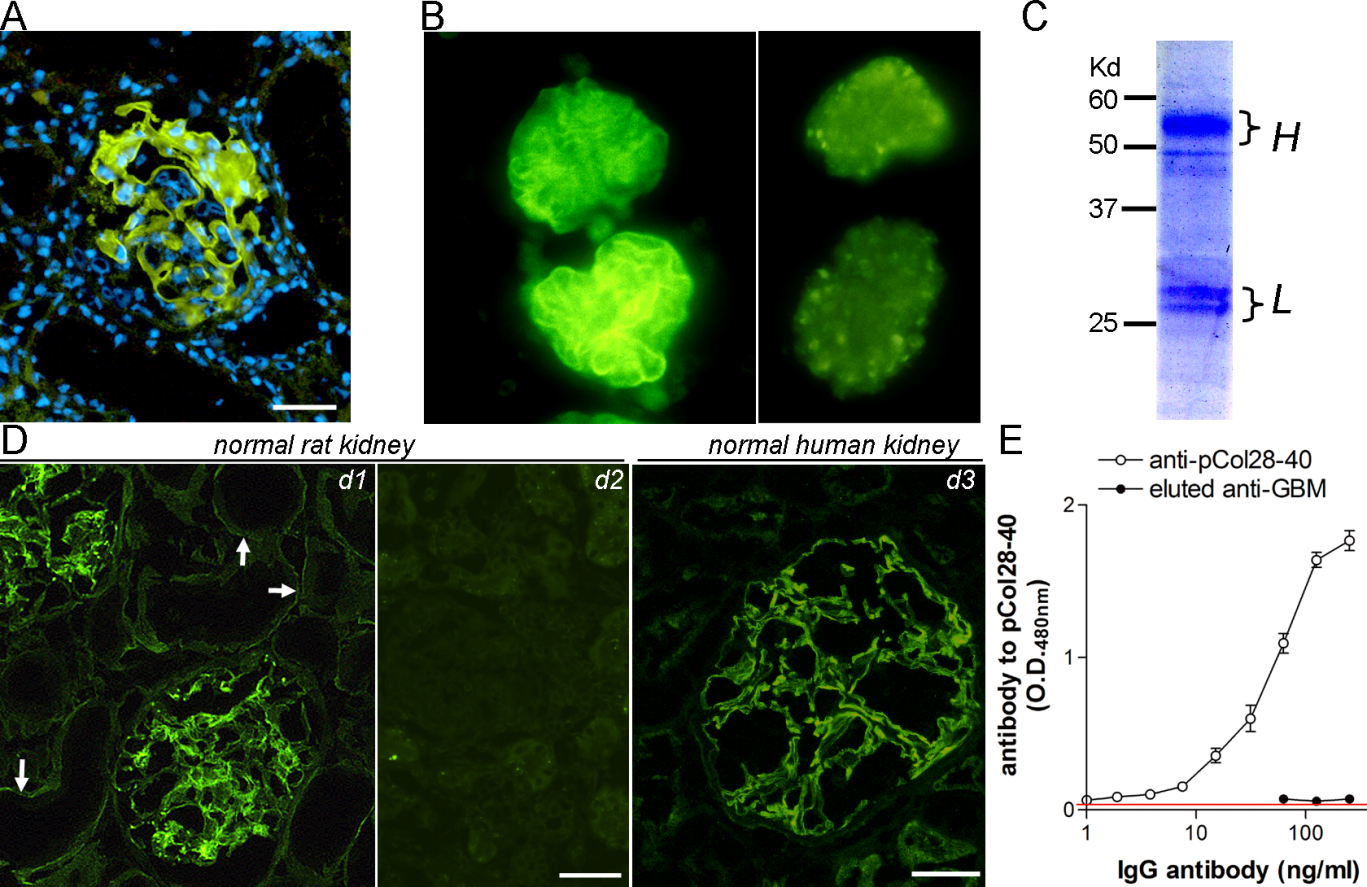
Isolation of anti-GBM autoantibodies from pCol4α3(28–40) immunized rats. (**A**) Direct immunofluorescence detection of IgG autoantibodies bound to GBM. (**B**) Immunofluorescence shows an isolated glomerulus before or after acid elution of GBM autoantibodies. Note disappearance of GBM-bound IgG. (**C**) SDS-PAGE analysis of the purity of eluted autoantibodies. Positions for IgG light and heavy chains are indicated. (**D**) Detection of anti-GBM activity in eluted autoantibodies by immunofluorescence on normal rat (*d1*) or human kidney (*d3*). Arrows show binding of eluted autoantibodies to tubular basement membrane. A control for normal rat kidney, which was stained by an irrelevant rat antibody and overexposed, is shown for comparison (*d2*). (**E**) ELISA detection of Ab activity to pCol4α3(28–40) in immunized mice. Note a high titer of anti-pCol4α3(28–40) activity in the serum of immunized rats but not in eluted autoantibodies. Bars = 50μm.

Thirty-four WKY rats were immunized with nephritogenic T cell epitope pCol4α3(28–40) and euthanized at day 41. As we have reported previously, no circulating anti-GBM autoantibodies were detectable in the rats. The kidneys were evaluated for anti-GBM autoantibodies by direct immunofluorescence. GBM-bound IgG autoantibodies were detected in all rats (Fig 1A). Glomeruli were then isolated and used for acid-based elution of autoantibodies (>15 rats/isolation). Direct immunofluorescence on a sample of isolated glomeruli before and after elution was performed. Vanishing of GBM-bound IgG suggested an effectiveness of the elution process (Fig 1B). SDS-PAGE analysis revealed two typical bands for the heavy chain and light chain of rat IgG (Fig 1C). Autoantibody activity and specificity in the eluate were further tested. We have reported previously that the GBM autoantibodies react to both rat and human native GBM (14). Immunofluorescence showed that the eluted autoantibodies reacted to both human and rat GBM (Fig 1D). The eluted autoantibodies also bound to tubular basement membrane in rats (TBM). However, the staining was much less intense (Fig 1D). As expected, ELISA showed that the autoantibodies did not react with immunizing peptide pCol4α3(28–40) (Fig 1E)[8]. The autoantibodies were then titered with human GBM by immunofluorescence due to its minimal background. Only those with a titer above 1:200-dilution were further used for screening by the random phage display peptide library.

### Identification of novel epitopes reactive to anti-GBM autoantibodies

**Fig 2:**
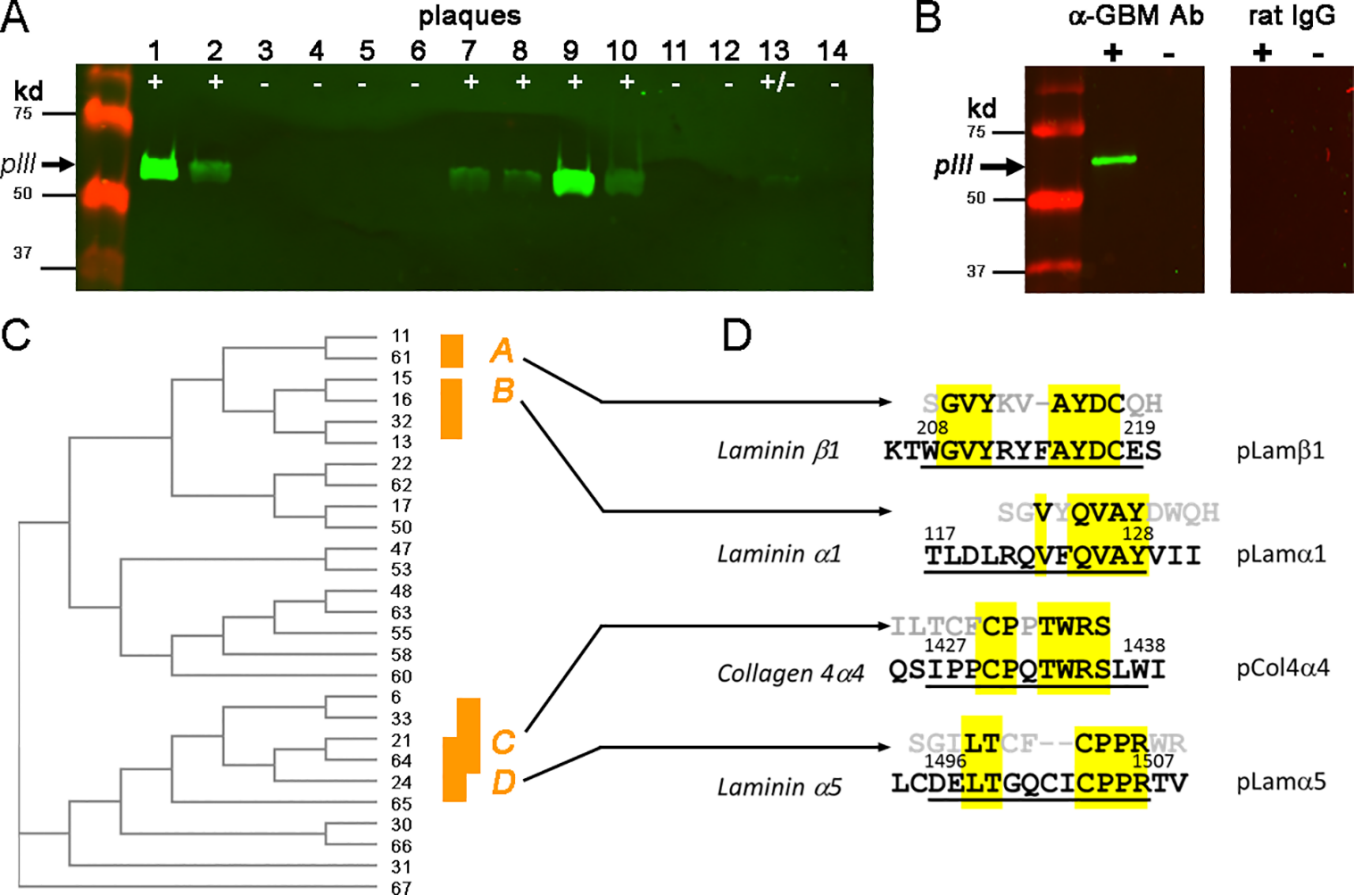
Identification of novel epitopes in GBM proteins recognized by eluted anti-GBM autoantibodies using random phage display peptide library. (**A**) Western blot screening of phage clones for their surface protein p*III* that positively reacted to GBM autoantibodies. Phage clones were from repeated panning by anti-GBM autoantibodies. (**B**) Confirmation of a representative clone that displayed positive reactivity to anti-GBM autoantibodies but not rat IgG by Western blot. Only one positive and one negative clone are shown. Note that the positive clone reacted to the Ab but not rat IgG. (**C**) Phylogeny tree for DNA sequences of 36 bp inserts from clones in which their pIII reacted to anti-GBM autoantibodies. Four clusters, indicated as A, B, C and D, are shown. (**D**) Comparison of a.a. sequences of GBM proteins with those deduced from A, B, C and D. Highlighted (yellow) letters indicate the identical residues or motif. Underlined letters are the identified a.a. sequences of GBM proteins (shown at the left) with their positions noted as superscript numbers. Flank a.a. residues were added for synthesis of 15-mer peptides as shown at the right.

After 3 sequential panning/amplification cycles, the captured phages were titered and used for testing their reactivity to eluted autoantibodies. A total of 60 phage plaques were randomly selected for isolation and amplification of phage clones. Proteins of amplified phages were screened for their specific binding to eluted autoantibodies with Western blot. Eluted autoantibodies recognized surface protein *p*III protein (65kD) on 25 phage clones (Fig 2A). On the other hand, these positively reactive phage clones showed no reactivity to normal rat IgG, suggesting specific affinity to autoantibodies rather than to the Fc region of IgG (Fig 2B). Phage DNA was isolated from positive phages and sequenced. Thirty-six nucleotide residues of the insert of *p*III gene were analyzed with a DNA alignment program. The resultant phylogenic tree for all inserts showed that the insert DNA sequences largely clustered into two separate groups (Fig 2C). We further chose two regions for each group. Translated amino acid residual sequences of the inserts formed four motifs, two for each cluster (A, B, C, and D)(Fig 2D). From those motifs, four peptides from basement membrane-related proteins Collagen 4α4(1427–1438), Lamininα1(117–128), Lamininβ1(208–219) and Lamininα5(1496–1507), respectively, were identified through gene bank searching (Fig 2D). Four 15-mer peptides were synthesized accordingly and named as pCol4α4, pLamα1, pLamβ1 and pLamα5, respectively (Fig 2D). These peptides were used for immunization of GN-susceptible WKY rats for evaluation of their pathogenicity.

### Novel Collagen 4α4-derived epitope induces glomerulonephritis

**Fig 3:**
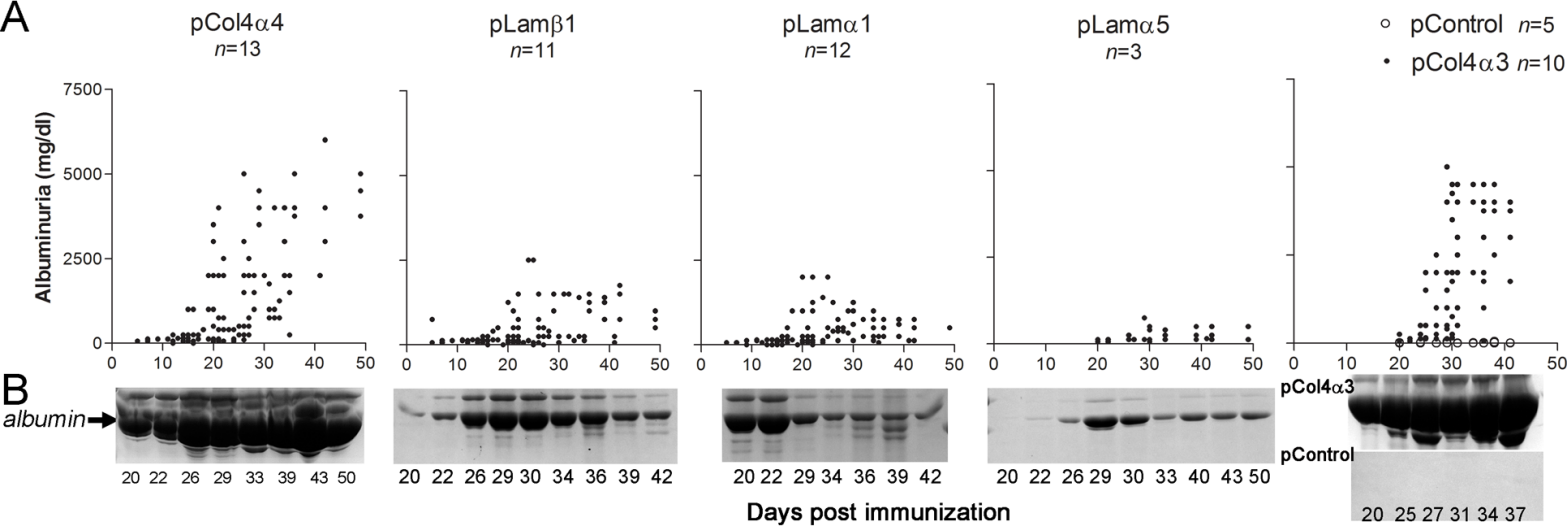
Development of albuminuria in rats post immunization with identified basement membrane peptides. (**A**) Time course of development of albuminuria. pCol4α3(28–40) or CFA-immunized rats were used as positive or negative controls. Albuminuria is expressed as mg/dl; each dot represents each rat at an indicated number of days. (**B**) SDS-PAGE shows albuminuria in a representative individual from each group at different time points after immunization.

**Fig 4:**
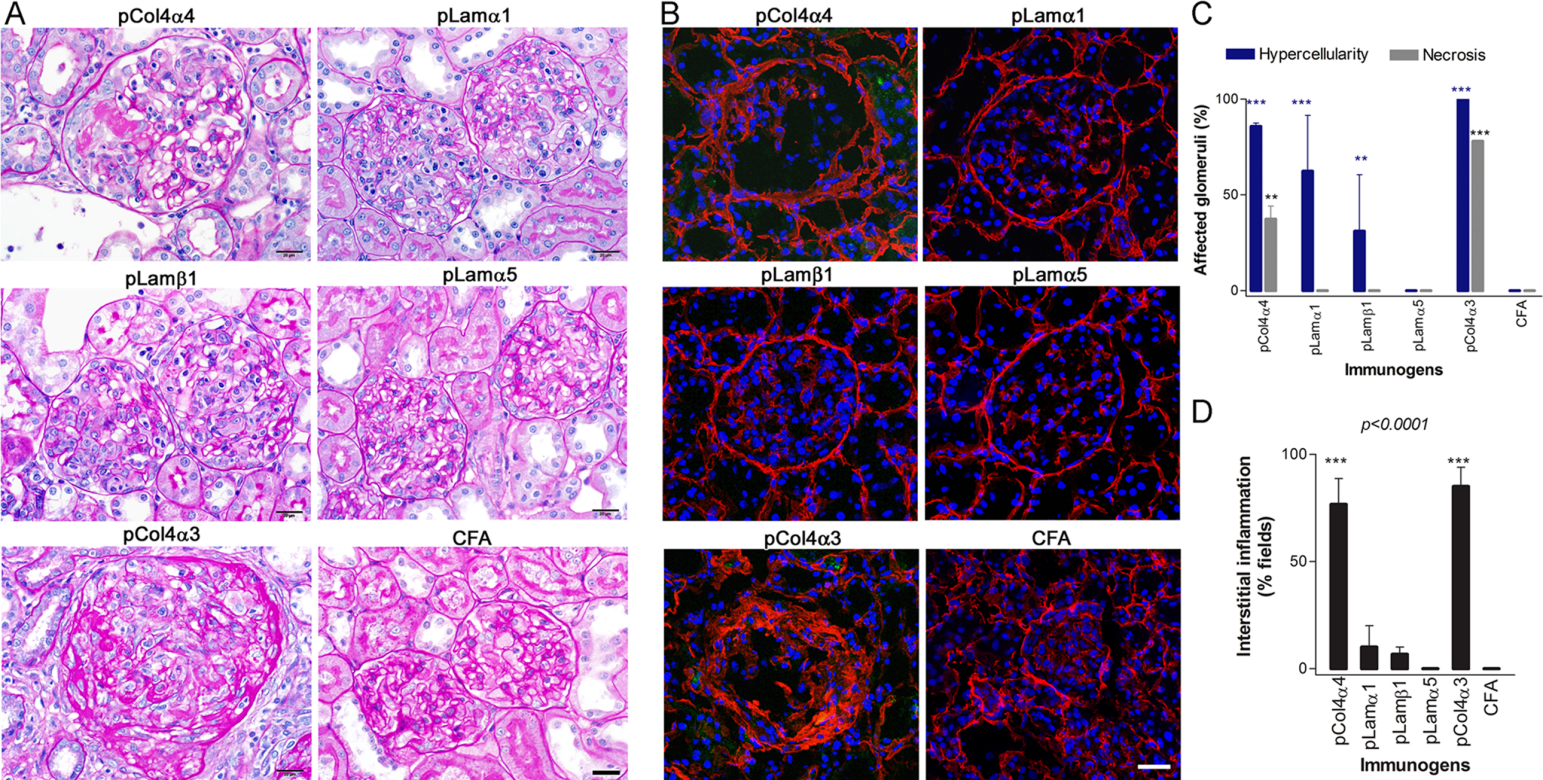
Glomerulonephritis in rats post immunization with identified new epitopes of basement membrane proteins. (**A**) Examples of the typical pathological changes present in kidneys of rats immunized with each peptide by PAS staining. *pCol4α4 (top*, *left)*: Hypercellular glomerulus with increased endocapillary mononuclear inflammatory cells and segmental, fibrinoid necrosis (weakly PAS-staining, pale pink) and epithelial proliferation (partial cellular crescent) located at 9:00 to 10:00. *pLamα1 (top*, *right)*: Two glomeruli with increased endocapillary mononuclear inflammatory cells, but no necrosis and no sclerosis. Glomeruli are smaller than those from rats immunized with pCol4α3 or pCol4α4. *pLamβ1 (middle*, *left)*: Glomeruli with increased endocapillary mononuclear inflammatory cells which appear similar to pLamα1, but are less numerous and likewise had no necrosis and no sclerosis. *pLamα5 (middle*, *right)*: Two essentially normal-appearing glomeruli with minimal mononuclear inflammatory cell infiltration. *pCol4α3 (bottom*, *left)*: glomerulus with sclerotic changes. Note the increased and disorganized deposition of strongly PAS-positive extracellular matrix proteins. *CFA (bottom*, *right)*: normal, control glomeruli. (**B**) Immunofluorescence shows the distribution pattern of collagen1α1 in a representative glomerulus for each group. Note that both pCol4α4 and control pCol4α3(28–40) group show extensive deposition of collagen1α1, suggestive of glomerular fibrosis. (**C**) Summary of pathological feature of glomeruli for each group. Bars = 50μm. (**D**) Summary for interstitial inflammation in each group.

Twelve WKY rats were immunized with pCol4α4. Rats immunized with pCol4α3(28–40) or CFA alone served as positive or negative controls, respectively. Albuminuria was monitored daily for each immunized rat. As expected, significant albuminuria was observed in pCol4α3(28–40)-immunized rats after day 20 and plateaued at day 30 (5,000mg/dl) (Fig 3A and 3B). Similar to pCol4α3(28–40)-immunized rats, those immunized with pCol4α4 began to develop albuminuria after day 15 to 20 (Fig 3A and 3B). In about half of the rats, albuminuria rapidly reached a plateau of 5,000mg/dl at day 30. Other rats eventually showed severe albuminuria after day 40. Rats immunized with pCol4α3(28–40) were euthanized at day 45 due to progressive worsening of their health. All other rats were sacrificed at day 50. We first compared renal pathology among those groups after H&E and PAS staining. As expected, rats immunized with pCol4α3(28–40) showed diffuse proliferative glomerulonephritis with segmental to circumferential, crescent-like necrosis in 72% of glomeruli (Fig 4). All glomeruli were diffusely and segmentally to globally hypercellular with increased numbers of glomerular cells and mononuclear inflammatory cells. Small lymphocytes appeared moderately numerous in glomerular capillaries. Both PAS stain and immunofluorescence on collagen 1α1 revealed segmental disruption of the glomerular basement membrane and early segmental sclerotic matrix changes affecting most of glomeruli (Fig 4A and 4B). The tubular interstitium was infiltrated diffusely by moderately numerous lymphocytes (Fig 4D). Proximal tubules had diffuse loss of PAS-positive brush borders, indicating acute epithelial injury, and focal, tubules showed early basement membrane wrinkling consistent with early tubular atrophy. GN with similar pathological features was observed in rats immunized with pCol4α4 but was less severe (Fig 4). Segmental to occasionally crescent-like necrosis was found in only 1% to 13% of glomeruli. PAS staining and immunofluorescence on collagen 1α1 revealed segmental disruption of the GBM and early segmental sclerotic matrix changes affecting many (27% to 50%) of the glomeruli (Fig 4A and 4B). The tubular interstitium was infiltrated diffusely by scattered to focally, moderately numerous, lymphocytes (Fig 4D). In contrast to pCol4α3(28–40) immunized rats, the proximal tubules had mostly preserved PAS-positive brush borders.

**Fig 5:**
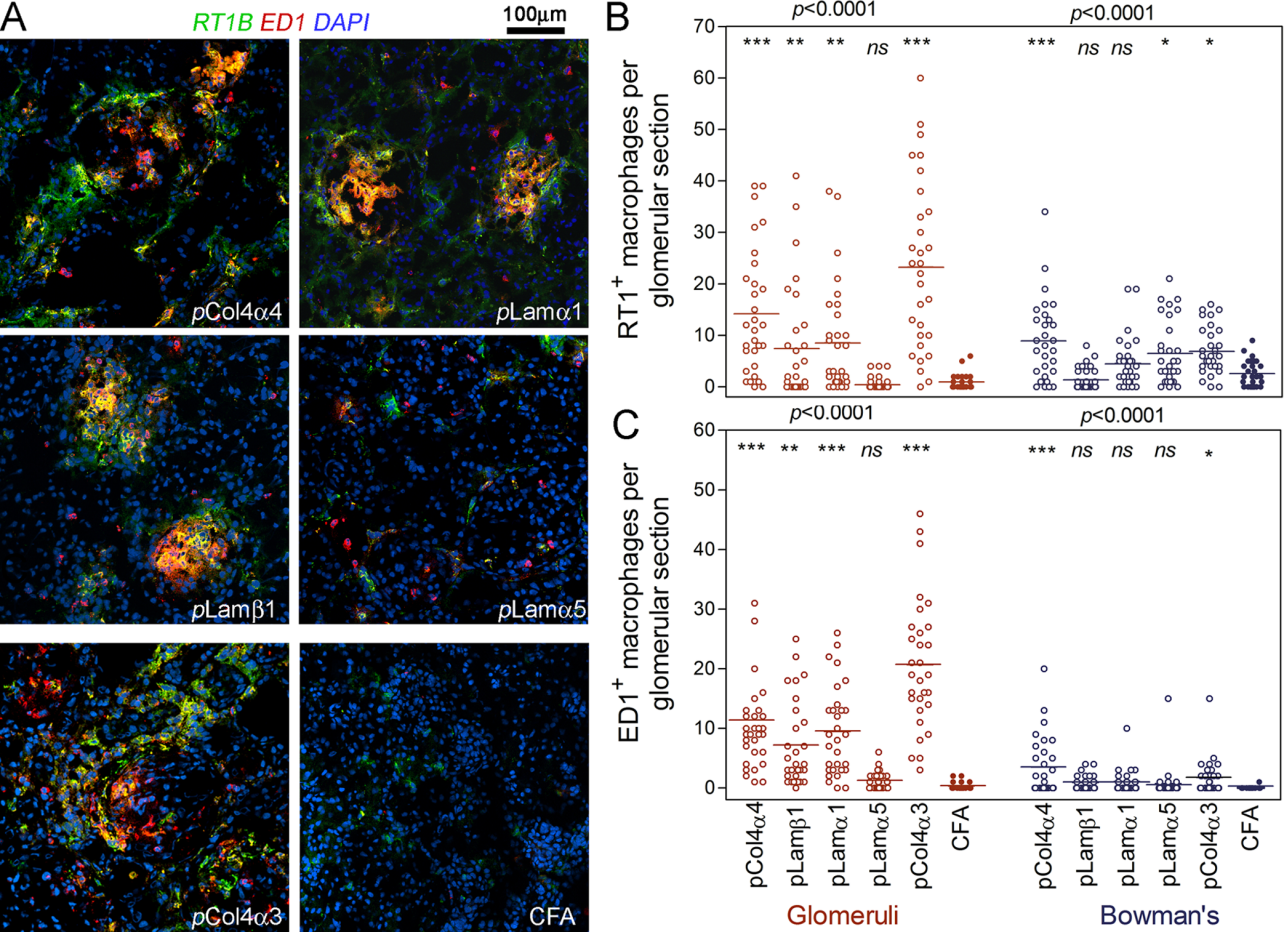
Quantitation of infiltrating macrophages in glomeruli of rats immunized with identified basement membrane peptides. (**A**) Immunofluorescence detection of subsets of macrophages in rats immunized with indicated peptides; note that three subsets of macrophages are identified, i.e. ED1^+^ (red), RT1B^+^ (green) and ED1^+^RT1B^+^ (yellow). (**B**) Statistical comparison of RT1B^+^ macrophages (including both RT1B^+^ and ED1^+^RT1B^+^ subsets) among rats immunized with various peptides. (**C**) Statistical comparison of ED1^+^ macrophages (including both ED1^+^ and ED1^+^RT1B^+^ subsets) among groups. Macrophages located within glomeruli or in Bowman’s capsule are counted separately. Total of 30 glomeruli from each group were randomly selected for counting macrophages.

We next quantitatively analyzed each subset of macrophages, and examined their distribution pattern of each group by immunofluorescence. As we have reported previously, in rats immunized with pCol4α3(28–40), that glomerular inflammation was gradually replaced by fibrotic tissue after day 35 [19]. Thus, the glomeruli of rats sacrificed at day 45 lacked infiltrating macrophages. Thus, we used kidneys of pCol4α3(28–40) immunized rats sampled at day 35. All three subsets of macrophages, i.e. RT1B^+^, ED1^+^ and RT1B^+^ED1^+^, were present among inflammatory cells infiltrating glomeruli in both the pCol4α4 group and the pCol4α3(28–40) control groups (Fig 5). The distribution patterns of these macrophageswere similar in the two groups; both being mainly located in glomeruli, and to a lesser degree in Bowman’s capsule (Fig 5A). Interestingly, the number of macrophages in Bowman’s capsule in the pCol4α4 group was higher than the pCol4α3(28–40) group (Fig 5B and 5C). In addition, both groups showed diffuse infiltration in the interstitium by macrophages. No infiltration was observed surrounding renal tubules.

### Pathogenic peptides from laminins induce inflammation in glomeruli but not in laminin-rich interstitial tissue

Rats immunized with either pLamα1 and pLamβ1 showed the same tendency: albuminuria peaked at 2000mg/dl at day 20 to 25, slightly declined after 30 days, and persisted until the end of experiments (Fig 3). On the other hand, pLamα5 immunization only induced very mild albuminuria (Fig 3). Rats immunized with pLamβ1 showed variable severe GN from almost normal to focal glomerular hypercellularity without necrosis (Fig 4A and 4C). These rats had minor tubulointerstitial inflammation or injury (Fig 4D). On the other hand, rats immunized with pLamα1 showed diffuse (79%-100%) glomerular hypercellularity with segmentally to globally hypercellular glomeruli but no necrosis (Fig 4A, 4B and 4C). The kidneys with more severe glomerular hypercellularity (100%) also had patchy tubulointerstitial inflammation (Fig 4D). Rats immunized with pLamα5 showed only mild GN with only slightly increased glomerular cellularity by comparison to CFA.

It is worthwhile to emphasize that, despite a wide distribution of laminins in the renal tissue, immunization with laminin-derived peptides led to more severe inflammation in glomeruli than in interstitial tissues. This was further demonstrated by immunofluorescence detection of macrophages. Immunofluorescence showed numerous infiltrating macrophages in glomeruli of both pLamα1 and pLamβ1 immunized rats with majority being ED1^+^RT1B^+^ type (Fig 5). Although rats immunized with pLamα5 did not display inflammation in H&E or PAS staining, a significant number of macrophages, especially the RT1B^+^ subset in Bowman’s capsules were detected in their glomeruli (Fig 5A and 5B). On the other hand, all three groups showed nearly no RT1B^+^ or RT1B^+^ED1^+^ macrophages in their interstitial tissue (Fig 5A). Only a few, if any, ED1^+^ macrophages were observed in the interstitial tissues (Fig 5A).

### Antibody and T cell response in immunized rats

**Fig 6:**
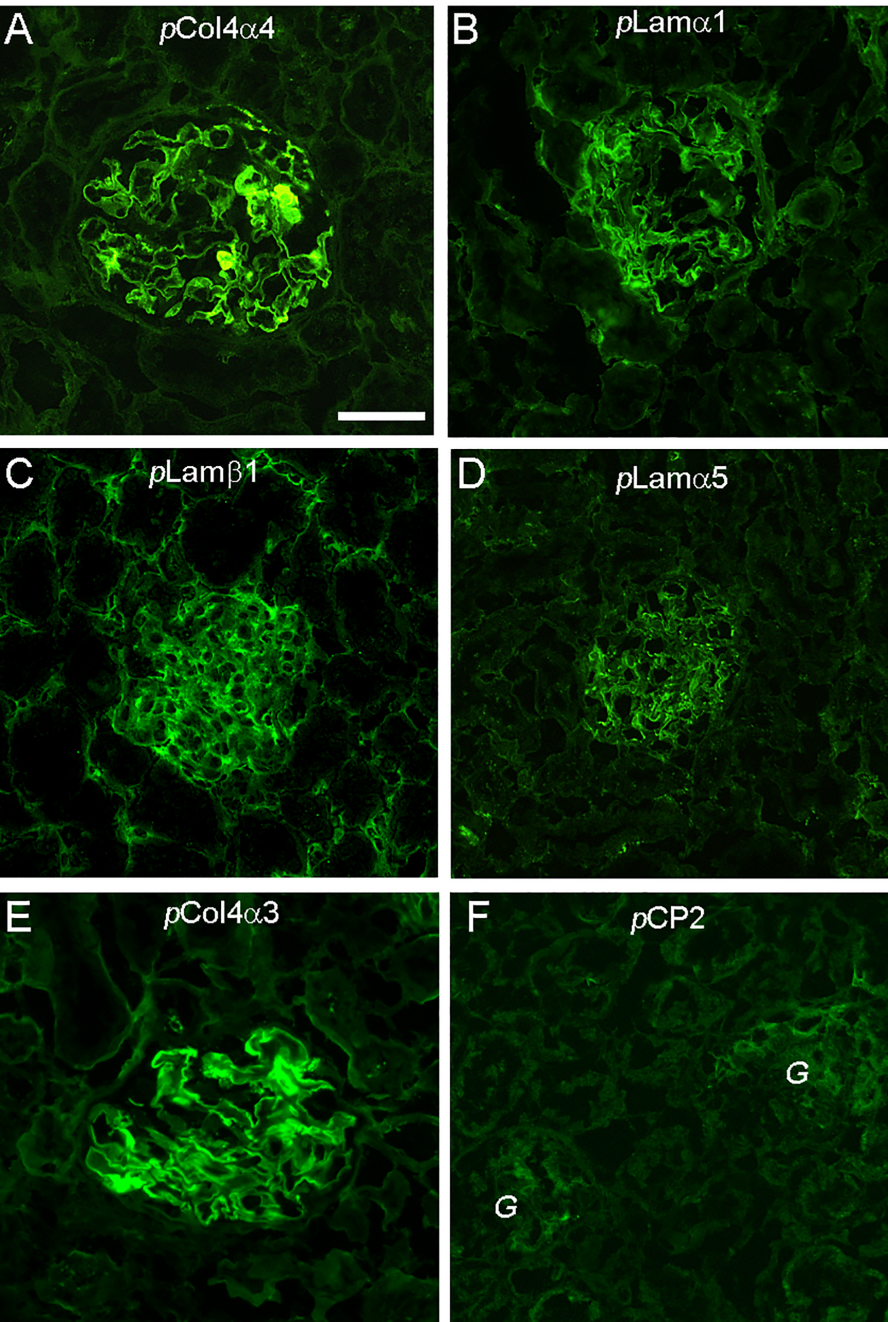
Autoantibody binding patterns in renal tissues in rats immunized with newly identified epitopes. Direct immunofluorescence detection of autoantibody in renal tissues in rats immunized with identified GBM peptides as indicated. *p*CP2, which is composed of a universal T epitope and murine ZP3 peptide [16], was used as an irrelevant control peptide; *G*, glomerulus. Bar = 50μm.

All tested peptides induced a strong T cell response regardless of their pathogenicity (data not shown). No or a very low level of circulating Abs to native GBM or TBM were detected in all immunized rats despite high titers of anti-peptide Abs of IgG isotype. However, immunofluorescence showed binding of Abs of IgG isotype in the renal tissue for all peptides, but each peptide showed a distinct binding pattern (Fig 6). In pCol4α4 immunized rats, typical linear binding of IgG autoantibody to GBM was observed, which was very similar to that in pCol(28–40)-immunized rats (Fig 6A and 6E). TBM-bound IgG was also detectable but in much lower intensity. Rats immunized with peptides pLamα1 and pLamβ1 showed binding of Ab in both glomeruli and interstitial tubules (Fig 6B and 6C). Linear binding of TBM was also observed, though it was much less intense (Fig 6B and 6C). In pLamα5-immunized rats, autoantibodies were detected in glomeruli and almost none was in the interstitial tissues (Fig 6D).

## Discussion

We have reported that a single nephritogenic T cell epitope not only induces anti-GBM GN, but also triggers production of autoantibodies to native B cell epitopes of GBM proteins beyond collagen4α3 [8]. We further demonstrated that the production of these anti-GBM autoantibodies is likely an autoimmunization by autoantigens leaked from glomerular damages due to auto-inflammation [18]. In this study using anti-GBM autoantibodies, we identified four potential B cell epitopes beyond the original autoantigen collagen4α3. Importantly, three of them were highly nephritogenic. Thus, we provide additional evidence supporting autoimmunization of leaked autoantigens and epitope spreading in this model. However, one may ask *why none of those identified B epitopes were from Col4α3NC1*, *since it contains multiple pathogenic peptides*? Our previous study identified multiple nephritogenic peptides in Col4α3NC1. Although those peptides contain linear B cell epitopes, they are peptide-specific epitope not shared with native epitopes present in the GBM [16]. Thus, it is reasonable that anti-GBM antibodies, which recognize only native GBM epitopes, are unable to fish out those peptides.

We have reported that passive transfer of pathogenic T cells induces glomerulonephritiswhich without triggering production of anti-GBM autoantibodies [20]. This suggests a lack of “epitope spreading” in the T cell transfer model. Interestingly, the pathology of GN in the T cell transfer model differs from that in an active immunization model with mainly T cells. One of the main differences between T cell transfer and active immunization is epitope spreading-induced autoantigen production. It will be interesting to ask how newly generated autoantibodies contribute to or modify pathogenesis of GN. We can further ask whether the pathogenesis of GN in an active immunization model is the sum of the pathogenesis caused by all T or B epitopes, including the original one and newly generated ones.

One important question regarding the consequence of inter-molecular epitope spreading is whether epitope spreading leads to “spread” of autoimmune inflammation beyond target tissues, because newly generated pathogenic epitopes are not necessary target tissue-specific. In the present study, three out of four newly identified pathogenic peptides were derived from laminins, which are common components of TBM [21, 22]. Furthermore, those proteins widely distribute in the basement membrane of various tissues. Interestingly, all three laminin peptides caused inflammation only in glomeruli, or to a much lesser degree in the interstitial tissues. For example, pLamα1 caused severe inflammation in more than 70% glomeruli but not in the interstitial tissues/tubules, despite the presence of a much larger quantity of laminin α1 in TBM than GBM [21]. Our preliminary study also showed absence of inflammation in several tissues such as the lungs of the immunized rats. TBM-bound IgG autoantibody to laminin can be detected in all rats immunized with laminin peptides, suggesting accessibility of TBM to autoantibody and probably to other immune molecules. Our histopathology and analysis on infiltrating leukocytes further showed that the glomerular inflammation was largely T cell-mediated as evidenced by the presence of numerous RT1B^+^ (MHC-II^+^) macrophages and T cells. Previous studies by other groups have shown that pathogenic T cells are able to cause interstitial inflammation [23]. Thus, the question becomes *Why did laminin-specific T cells fail to cause inflammation in laminin-abundant renal interstitial tissues or in other tissues*? This question still remains to be explored. However, a possible explanation is different susceptibility to autoimmune inflammation for each tissue. In other words, glomeruli probably are more susceptible to inflammation than the surrounding tissues. We have reported a critical role of special tissue environment in controlling autoimmune inflammation. A target tissue, which has recovered from a previous autoimmune inflammation, becomes resistant to the same type of autoimmune inflammation [24]. A similar phenomenon has been described in the EAE model as well [25]. Thus, it is possible that surrounding interstitial tissues may have “learned” the resistance to inflammation from nearby glomerular inflammation. One may argue why interstitial inflammation was observed in glomerulus-specific autoantigens (i.e. collagen 4α3). Careful examination revealed that such inflammation was closely associated with glomeruli with almost all inflammatory cells close to or surrounding Bowman’s capsule but not TBM. Thus, the interstitium may not be a target tissue in this case, and the inflammation is of a collateral nature. In summary, our results revealed that even when newly generated pathogenic epitopes were present in the autoantigens shared between the target and surrounding tissues, autoimmune inflammation did not extend beyond the target tissues. Our discovery may reveal a critical protective mechanism necessary for the prevention of the spread of inflammation beyond the target tissue, during either autoimmune pathogenesis or other events such as infections.

There are a few additional interesting factors related to those newly identified epitopes. *First*, our results suggest involvement of a potentially large number of B cell epitopes on GBM components beyond collagen4α3 in epitope spreading. Since this has been described in several autoimmune diseases models, it is not an unexpected finding [4–9]. However, four peptides were identified after screening only a very small portion of positive phage plaques. Thus, it is predictable that many other epitopes may also have been generated during pathogenesis of GN. On the other hand, all epitopes were derived from proteins in GBM, suggesting that inter-molecular epitope spreading may limit to closely associated proteins, an observation supported by several previous studies [6, 9]. *Second*, although new epitopes were identified through screening with anti-GBM autoantibody, all of them contained nested T cell epitope(s), since rats immunized with these peptides elicited T cell response, as evidenced by production of a high titer of IgG type Abs post immunization. Interestingly, despite a high titer, the circulating Ab showed a strong reactivity to the peptides but not to native basement membranes. Thus, the original B cell epitopes on the autoantigens are most likely 3-D and not the linear conformation. Since the majority of Abs were peptide-specific, the T cell epitope(s) in the peptides are most likely to be responsible for GN. If that is the case, this raises the question, *does a B cell epitope of an autoantigen always contains a pathogenic T cell epitope*, *or is our discovery merely a coincidence*? Numerous mapping studies have revealed a low frequency of pathogenic T cells epitopes on a given protein autoantigen [26–28]. For example, only three among many peptides, which cover collagen 4α3NC1, show nephritogenicity [16]. Surprisingly, all four newly identified B cell epitopes contained pathogenic T cell epitopes in this study. This high frequency suggests that co-existence of B and T cell epitope may be common. Several other studies also showed similar results. A B cell epitope, which was originally identified by an Ab to an ovarian antigen, contains a pathogenic T cell epitope [29]. This phenomenon should be further explored.

It remains unclear if inter-molecular epitope spreading is a universal phenomenon in autoimmune glomerulonephritis in animal models or in human Goodpasture’s syndrome. Rat models for anti-GBM disease largely show inter-molecular spreading [8, 9]. Early studies by Hudson’s group has demonstrated that almost all autoantibodies in patients with Goodpasture’s syndrome were directed against the NC1 domain with no particular antibody response to laminin, suggesting the absence of inter-molecular epitope spreading in this disease [30]. On the other hand, indirect evidence from recent studies supported occurrence of inter-molecular epitope spreading in this disease, since some autoantibodies did not react with known epitopes in collagen 4α3 [31]. In humanized mouse models, anti-GBM antibodies are associated with pathogenicity of T cell epitopes due to different MHC restriction, but not circulating antibody to the peptide, suggesting the occurrence of epitope spreading [32]. It would be interesting to ask if MHC II restriction or specificity of an original pathogenic peptide may determine the occurrence of epitope spreading. From this point of view, our model may be a useful tool, since several pathogenic T epitopes have been identified in Col4α3NC1 with known MHC restriction [16, 33]. Further investigation will be necessary to determine whether these T epitopes also trigger epitope spreading.

## Abbreviations

EAE: experimental autoimmune encephalomyelitis
GBM: glomerular basement membrane
GN: glomerulonephritis
TBM: tubular basement membrane
WKY: Wistar Kyoto
